# Evaluation of polymerization shrinkage stress and cuspal strain in natural and typodont teeth

**DOI:** 10.1590/1807-3107bor-2024.vol38.0061

**Published:** 2024-08-05

**Authors:** Luiza Santos CARDOSO, Amanda Alves de OLIVEIRA, Gabrielly D’Paula Muniz BARBOSA, Maria Luisa Prazeres RIBEIRO, Tainah Costa FIRMIANO, Crisnicaw VERÍSSIMO

**Affiliations:** (a) Universidade Federal de Goiás – UFG, Dental School, Department, Goiânia, GO, Brazil.

**Keywords:** Dental Stress Analysis, Polymerization, Finite Element Analysis, Composite Resin

## Abstract

To evaluate the polymerization shrinkage stress and cuspal strain (CS) generated in an artificial (typodont) and in a natural tooth using different resin composites. Twenty artificial and 20 extracted natural molars were selected. Each tooth was prepared with a 4x4 mm MOD cavity. The natural and typodont teeth were divided into four experimental groups (n=10), according to the resin composite used: Filtek Z100 (3M Oral Care) and Beautifil II LS (Shofu Dental). The cavities were filled using two horizontal increments and the CS (µS) was measured by the strain gauge method. Samples were sectioned into stick-shaped specimens and the bond strength (BS) (MPa) was evaluated using a microtensile BS test. Shrinkage stress and CS were analyzed using 3D finite element analysis. No difference was found between the type of teeth for the CS as shown by the pooled averages: Natural tooth: 541.2 A; Typodont model: 591.4 A. Filtek Z100 CS values were higher than those obtained for Beautifil II LS, regardless of the type of teeth. No statistical difference was found for the BS data. Adhesive failures were more prevalent (79.9%). High shrinkage stress values were observed for Filtek Z100 resin, regardless of tooth type. The CS of typodont teeth showed a shrinkage stress effect, generated during restoration, equivalent to that of natural teeth.

## Introduction

During the placement of direct posterior restorations using resin composite, the polymerization process results in volumetric shrinkage, which is an inherent characteristic of resin-based restorative materials that occurs as a result of the formation of a polymeric network during the polymerization reaction.^1-3^ This factor generates shrinkage stress, compromising the integrity of the restoration.^4^

The resin composite adhered onto cavity walls causes cuspal flexure and strain, and it is related to shrinkage stress.^1^ These phenomena have been associated with the intrinsic characteristics of the resin composite, such as post-gel shrinkage and elastic modulus, and with other factors, e.g., filling technique, cavity design, light-curing process, and operator’s skill.^5-7^ Shrinkage can generate stresses that have been associated with clinical problems such as cuspal flexure and strain, enamel crack propagation, postoperative sensitivity, gaps, and secondary decay.^8-11^ The residual shrinkage stress may compromise bonding.^4^ Another factor often associated with high residual shrinkage stress is marginal staining.^12,13^

Finite element analysis is the most comprehensive method to calculate this complex stress and strain condition generated by resin composite shrinkage .^14^ Furthermore, cuspal strain, generated during the restoration, can also be measured by the strain gauge method.^15^ The strain gauge is an electrical resistance that can be attached to the external tooth surface to monitor and collect, through a data acquisition device, the strain developed during the restoration.^15,16^

The application of natural teeth in dental research to measure cuspal flexure and strain during the placement of a resin composite restoration is a common practice, but it is susceptible to variation in the anatomical aspects of the dental elements and relies on the complex acquisition of extracted teeth. The use of human teeth without proper sample standardization may jeopardize the accuracy of the results and the validity of the study.^17^ To solve this problem, the geometric standardization offered by artificial teeth (typodont) is promising and an alternative, as shown by Enochs et al.^17^ Such geometric standardization of the cavity makes it easier to study the stresses and strains generated during restoration with different commercial composites. However, the promising results of this study evaluated only the cuspal flexure generated by different composites. Since the development of new resin composites, monomers, and restorative techniques is continuous in dental research, the application of the typodont model to different shrinkage analyses such as CS by the strain gauge method is pertinent. Therefore, the aim of this study was to evaluate whether the use of artificial teeth (typodont) is an effective alternative to natural teeth for the analyses of the effects of polymerization shrinkage and CS. The null hypothesis tested was that the CS and bond strength (BS) values would not be affected by the type of tooth (natural or typodont) and type of resin composite (conventional or low-shrinkage).

## Methods

### Composite mechanical characterization

Filtek Z100 (3M Oral Care) and Beautifil II LS (Shofu Dental) were chosen for the experimental tests (Table 1) because they express different shrinkage behaviors: high (Filtek Z100)^18^ and low shrinkage (Beautifil II LS).

**Table 1:** Composition of the resin composites used.

Material	Batch	Shade	Classification	Organic matrix	Filler type	% Wt	F% Vol
Filtek Z100	2028300472	A2	Microhybrid	Bis-GMA, TEGDMA	Zirconia and silica	85	66
Beautifil II LS	12036	A2	Nanohybrid, Bioactive Composite (Giomer)	Bis-GMA, TEGDMA	Multifunctional glass and S-PRG filler based on fluoroboralumino silicate glass	83.3	68.6

### Post-gel shrinkage

Post-gel shrinkage was determined using the strain gauge method.^15^ The restorative material (n = 10) was shaped into a hemisphere on a biaxial strain gauge (PA-06-060 TH-120L, Excel Sensores). The composite was light-cured for 40 seconds using RadiiCal (SDI). The strain gauge was connected to a data acquisition device (ADS1800, Lynx Electronic Technology). The mean shrinkage strain was converted into linear volumetric post-gel shrinkage (%).

### Elastic modulus determination

The resin composite (n = 10) was placed in a circular silicon mold, 2 mm deep and with a 4 mm diameter, and was light-cured for 20 seconds. Five Knoop indentations were made (HMV-G 21DT Shimadzu) in each specimen.^19^

### Compressive tensile and diametral tensile strengths

For compressive strength (n = 10), the specimens were fabricated with a matrix of metal (3 mm-diameter, 6 mm-height). For the diametral tensile strength (n = 10), the specimens were fabricated using a silicone mold with 6 mm-diameter and 3 mm-height. The specimens were subjected to tests on a universal testing machine (Instron 5965).

### Tooth selection and cavity preparation

Twenty extracted intact caries-free natural mandibular molars were used after approval by the local Research Ethics Committee (Process: 42164821.8.0000.5083). The molars had a mesiodistal width of 11 mm and a buccolingual width of 10 mm, with a maximum deviation of 10% from the determined mean.

For typodont selection, the Knoop microhardness test was performed to determine the elastic modulus of three different commercial brands: MOM (Manequins Odontológicos Marília Ltda) with 32.0 GPa; P-oclusal (P-Oclusal Prod. Odont. Ltda) with 2.53 GPa; and Prodens (Prodens Prod. Odont. Ltda) with 2.09 GPa. The MOM typodont was chosen because it has a higher elastic modulus. This typodont has Araldite MY 750 epoxy resin in its composition. Furthermore, the anatomy is similar to the lower first molars and average dimensions of 10.8 mm on the mesiodistal surface and 10.2 mm on the buccolingual surface used for size standardization of the selected natural teeth.

Class II cavities (mesio-occlusal-distal) were prepared with diamond burs #3099 (KG Sorensen), with 4 mm deep from the highest cusp and 4 mm wide (buccolingual).

### Typodont and natural teeth bond procedures

Artificial teeth differ from natural teeth in their composition; therefore, they do not enable the demineralization that occurs in enamel and dentin hybridization . As an alternative, a 50 μm aluminum oxide microjet was used for 30 seconds (Bio-Art) to promote micromechanical retention and favor the adhesion process. The enamel of the natural teeth was etched with 37% phosphoric acid (Condac, FGM). A universal adhesive system (Single Bond Universal, 3M Oral Care) was applied.

### Restorative filling technique

The artificial and natural teeth were randomly divided into four groups (n = 10) according to the composite and type of tooth: Filtek Z100 and natural teeth, Filtek Z100 and typodont, Beautifil II LS and natural teeth, and Beautifil II LS and typodont. The resin composites were placed using two horizontal increments in the prepared cavity joining three walls (two opposite surrounding walls and one at the bottom). Each increment of resin composite was light-cured for 20 seconds (RadiiCal, SDI) according to the manufacturer’s instructions. This device has a 10-mm active tip, adequate irradiance (radiant exposure fixed at 1,200 mW/cm2), and a collimated light beam that maintains the adequate intensity for the dimension adopted.^20^

### Cuspal strain during the restorative procedure

The strain gauges were bonded with cyanoacrylate adhesive (Super Bonder, Loctite) in the cervical region of the buccal and lingual external surfaces. The strain gauges were connected to a data acquisition device (ADS1800, Lynx Electronic Technology) to record strains (µs). The teeth were restored with the different composites (n = 10). The frequency of 4 Hz was used to collect the CS data during the restorative procedure and was monitored for 10 minutes after curing the last increment through the data analysis software (AqDados 7.05 and AqAnalisys, Lynx Electronic Technology).

### Bond strength

For the test, 249 specimens of 40 teeth were obtained, and pretest failures were excluded from the sample. The cuts were performed perpendicularly to the adhesive surface, obtaining stick-shaped specimens with a cross-sectional area of approximately 1 mm^2^. Each stick-shaped specimen was actively gripped onto a Geraldeli’s device (Od04-Plus, Odeme Dental Research). The specimens were pulled up to the moment of fracture at a constant speed of 0.5 mm/min and the maximum load value at the moment of rupture was recorded for each specimen.^21^ The failure mode was classified according to the following criteria:^22,23^ adhesive; cohesive in resin composite; cohesive in natural tooth; cohesive in typodont.

### Residual shrinkage stress – 3D finite element analysis

**Figure 1:**
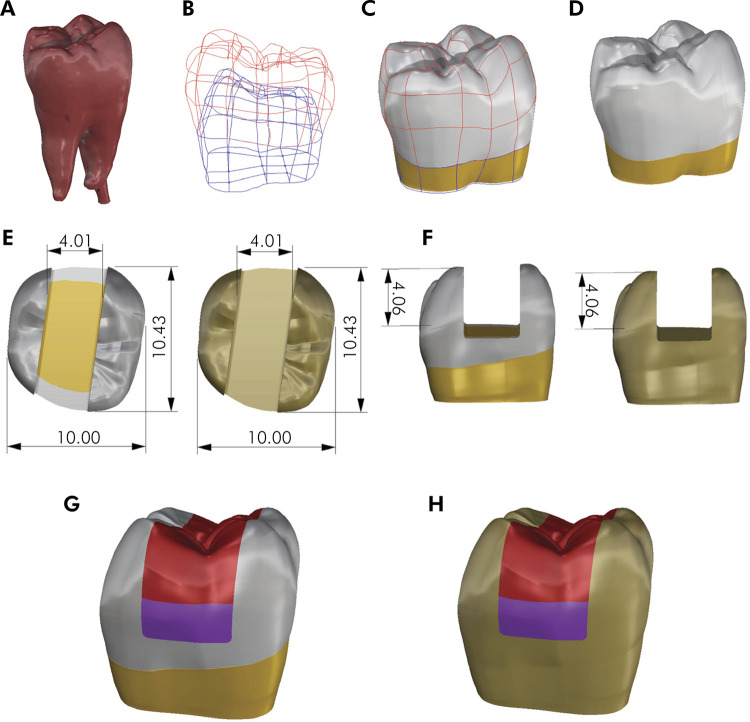
Bio-CAD three-dimensional modeling protocol.

A three-dimensional molar model was created using the Bio-CAD modeling protocol from an intact mandibular human molar using computer-assisted design (CAD) software (Rhinoceros 3D 5.0). The tooth was scanned and the external contour was saved in stereolithography (STL) file (Figure 1A). The models were created using NURBS surfaces (non-uniform rational Bezier spline), using lines (Figures 1B, C). The typodont model was created based on the external geometry of the natural tooth (Figure 1D). The 4x4 mm MOD preparation was simulated with the same dimensions of the experimental test (Figures 1E, F).

A. STL file of scanned mandibular third molar; B. Polylines; C. Polylines and surfaces created in the STL file; D. Enamel, dentin surface of the molar model; E. Occlusal view and dimensions of the MOD class II preparation (natural tooth and typodont); F. Proximal view and dimensions of the preparation (natural tooth and typodont); G. Natural tooth filled with two horizontal increments; H. Typodont model filled with two horizontal increments.

The models were exported as Standard for the Exchange of Product Data (STEP) files to a preprocessing program known as Patran Software 2010, (MSC Software Corporation) for the creation of a finite element mesh of each structure using a four-noded solid tetrahedral element. The mesh of each structure was exported to the FEA program (MSC. Marc and MSC. Mentat, MSC Software Corporation). Boundary conditions, consisting of constrained nodal displacement in the x, y, and z dimensions on the bottom surface of the model, were applied. Polymerization shrinkage was simulated by thermal analogy (steady thermal analysis). The notional temperature was reduced by 1 ºC, while the post-gel shrinkage strain was entered as the coefficient of linear thermal expansion.^24^ Each resin composite increment was activated in the FEA software following the same sequence of the restoration filling technique employed in the experimental test (Figures 1G, H). All material responses were considered linear-elastic and isotropic, and all interfaces between materials were bonded. Material properties applied in the finite element analysis are shown in Table 2. Dentin was characterized based on the study by Rees; Jacobsen; Hickman,^25^ whereas enamel was characterized according to the study by Zarone et al.^26^ The estimation of the value of the Poisson ratio of the resin composites was based on the Versluis^9^ study. The residual shrinkage stresses (MPa) were evaluated by modified Von Mises equivalent stress,^27^ calculated using the listed compressive and tensile strength values.^28^

**Table 2:** Compressive and diametral tensile strengths, elastic modulus, Poisson ratio, and post-gel shrinkage applied in the finite element analysis.

Material	Compressive strength (MPa)	Diametral tensile strength (MPa)	Elastic modulus (GPa)	Poisson ratio	Linear post-gel shrinkage (%)
Beautifil II LS	305.69 (45.51)*	35.68 (8.21)*	9.5 (0.7)*	0.24	0.13102*
Filtek Z100^28^	176.3 (15.4)	51.2 (6.5)	20.8 (1.15)*	0.24	0.32151*
Typodont	125.0**	65.0**	32.0**	0.24	
Dentin^25^	297.0	98.0	18.3	0.31	
Enamel^26^	384.0	10.3	84.0	0.33	

*Experimentally determined in this study; ^**^Data provided by the manufacturer (MOM, Brazil).

### Statistical analysis

The averaged buccal and lingual CS values obtained in the CS experiment and bond strength analysis were tested for normal distribution (Shapiro-Wilk, p > 0.05) and equality of variances (Brown-Forsythe, p > 0.05). Two-way ANOVA was performed for the CS and BS. The pairwise multiple comparison procedure was performed using Tukey’s test. The significance level was 0.05 and the analyses were performed using Sigma Plot statistical software package (version 14, Systat Software Inc.).

## Results

### Cuspal strain during the restorative procedure

The averaged buccal and lingual CS, and the pooled averaged values after 10 minutes with the strain gauges are shown in Table 3. Two-way ANOVA indicated that the resin composite isolated factor (conventional or low-shrinkage) was significant (p < 0.001). The type of tooth (natural or typodont) isolated factor (p = 0.449) and the interaction between the type of tooth and resin composite were not significant (p = 0.064). No difference was found in CS between the types of tooth (natural or typodont). Filtek Z100 showed higher values of CS than the Beautifil II LS, regardless of the type of tooth (natural or typodont).

**Table 3:** Mean (SD) of the averaged buccal and lingual cuspal strain values by the strain gauge method.

Variable	Natural tooth	Typodont model	Pooled average
Filtek Z100	711.0 (173.6)	886.3 (308.1)	798.7 A
Beautifil II LS	371.5 (163.7)	296.4 (138.9)	333.4 B
Pooled average	541.2 A	591.4 A	

**Figure 2:**
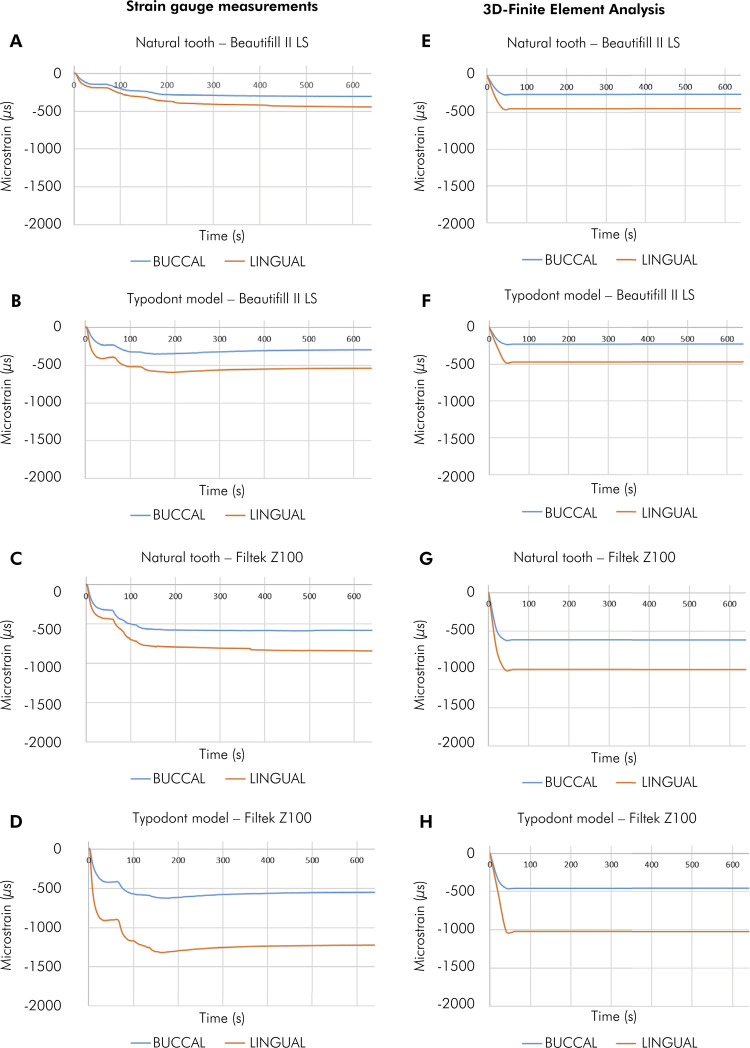
Cuspal strain by the strain gauge measurements.

The history plot of CS during the restoration for the experimental strain gauge measurements and finite element analysis is shown in Figure 2. The analysis of the strain curves showed that the Filtek Z100 CS values were higher than those of the Beautifil II LS, regardless of the type of tooth (natural or typodont). The lingual cusp showed higher strain values than the buccal cusp. The experimental curves of CS exhibited a similar pattern to that of the finite element analysis.

A. Natural tooth - Beautifil II LS; B. Typodont - Beautifil II LS; C. Natural tooth - Filtek Z100; D. Typodont - Filtek Z100. Cuspal strain by 3D finite element analysis: E. Natural tooth - Beautifil II LS; F. Typodont -Beautifil II LS; G. Natural tooth - Filtek Z100; H. Typodont - Filtek Z100.

### Bond strength analysis

**Figure 3:**
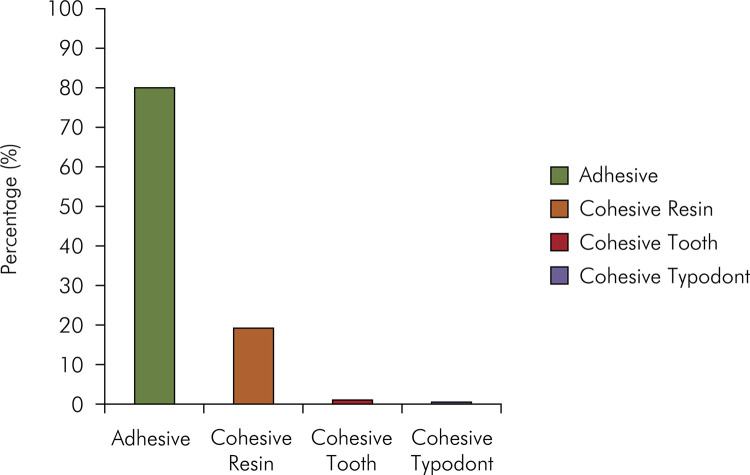
Bond strength failure mode.

The mean BS and the pooled averaged values are shown in Table 4. Two-way ANOVA indicated that the resin composite isolated factor (conventional or low-shrinkage) (p = 0.752), type of tooth (natural or typodont) (p=.055), as well as the interaction between them, were not significant (p = 0.328). The failure mode analysis is shown in Figure 3. Adhesive failure was the most prevalent failure mode (79.9%), followed by cohesive failure in resin (18.9%), cohesive failure in tooth (0.8%), and cohesive failure in typodont (0.4%).

**Table 4:** Mean (SD) of the bond strength values by the microtensile test.

Variable	Natural tooth	Typodont model	Pooled average
Filtek Z100	26.5 (4.7)	28.6 (6.9)	27.5 A
Beautifil II LS	25.1 (5.9)	31.3 (3.5)	28.2 A
Pooled average	25.8 A	29.9 A	

### Residual shrinkage stress – 3D finite element analysis

**Figure 4:**
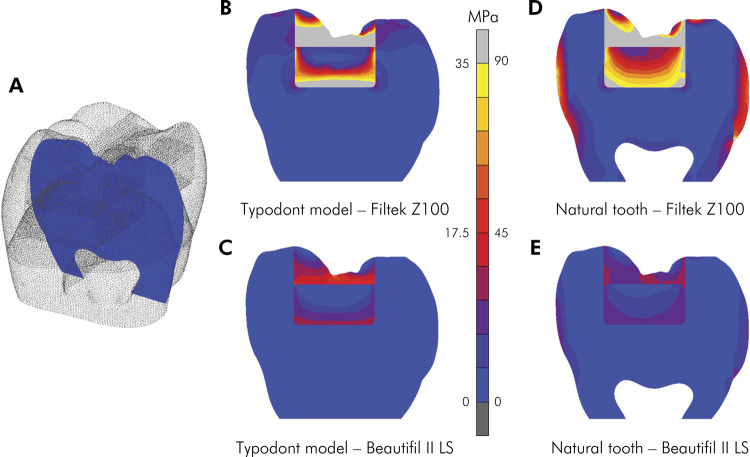
Modified Von Mises shrinkage stress (MPa) distributions on the cross-sectional plane.

The modified Von Mises shrinkage stress distributions (MPa) on the cross-sectional plane (Figure 4A) are shown in Figure 4. The stresses can be visualized in the linear color scale, where blue indicates the lowest stress values and yellow and light gray the highest stress values.

A. Selected cross-sectional plane; B. Typodont model – Filtek Z100; C. Typodont model – Beautifil II LS; D. Different stress scale for typodont and natural models; E. Natural tooth – Filtek Z100; F. Natural tooth – Beautifil II LS.

Stress distributions in typodont restorations and at restoration interfaces are similar. The main difference is that the stresses are lower in the typodont model (Figures 4B, 4C) (note the different stress scale for typodont and natural models). In the natural tooth model, stresses are also concentrated in the buccal and lingual enamel area (Figures 4D, 4E). The higher residual shrinkage stress values were observed for Filtek Z100, regardless of the type of tooth (typodont (Figure 4B) or natural (Figure 4D).

## Discussion

The use of typodont for shrinkage analysis was firstly reported by Enochs et al.^17^ in 2018. The authors showed the typodont was a viable option as a substitute for natural teeth for evaluation of cuspal flexure and to study the effects of polymerization shrinkage stress. The use of typodont applied in the cuspal flexure methodology was proposed to replace the use of human teeth with cuspal flexure tests given that their acquisition became very difficult. Moreover, the use of standardized typodont teeth also facilitates the execution of the method. However, the promising results shown by Enochs^17^ were for one specific methodology: cuspal flexure calculated by the optical method. The effects of shrinkage stresses developed by resin composites can also indirectly be assessed using the strain gauge method. In this method, a strain gauge with an electrical resistance is attached to the tooth and the deformation and strain generated during the restorative procedure can be acquired using a data acquisition device. Therefore, the aim of this study was to evaluate whether artificial teeth (typodont) are an effective alternative to natural teeth in the analysis of polymerization shrinkage and CS by the strain gauge method.

The null hypothesis tested in this study was that CS and BS values would not be affected by the type of tooth (natural or typodont) and by the type of resin composite (conventional or low-shrinkage). CS and BS values were not affected by the type of tooth (natural or typodont). However, the type of resin composite (conventional or low-shrinkage) affected CS values. Therefore, the null hypothesis (h0) was partially accepted.

Two different resin composites were tested in this study. The post-gel shrinkage values of the resin composites were determined in this study during the characterization of the composites. Filtek Z100 showed higher linear post-gel shrinkage values (0.32%), corroborating the findings of Oliveira et al.^18^ On the other hand, Beautifil II LS showed a lower value of linear post-gel shrinkage (0.13%), which is in accordance with the data presented by manufacturers, who claim that it is a low-shrinkage material. The use of two different composites with very contrasting post-gel shrinkage values was important to observe the effects on the different substrates (typodont or natural tooth). Two horizontal increments were used for comparison of our results with those obtained by Enochs et al.^17^ In addition, values of elastic modulus and tensile and compressive strengths of each resin were determined. The results of our study show that typodont teeth were able to express the contrast of the polymerization shrinkage effects of different resin composites, one with high shrinkage and the other one with low shrinkage, similarly to the natural tooth, considering that there was no statistical significance regarding the type of tooth (p<.001). This contrast is also related to the difference in elastic modulus values, as the elastic modulus of Filtek Z100 (20.8 GPa) is higher than that of Beautifil II LS (9.5 GPa). Corroborating this finding, the strain curves collected by the experimental CS method and finite element analysis were very similar. Comparing the pattern of history plot of CS during the restoration for the experimental strain gauge measurements and finite element analysis, the results of our study show that the typodont was able to express the contrast of the polymerization shrinkage effects of the different resins in a similar way to the natural tooth, considering that there was no statistical significance regarding the type of tooth (p < 0.001), thereby validating our results.

Obviously, there is no stress and strain development if the composite is not bonded to the tooth structure or substrate.^13^ To assess the adhesion between the composite and the typodont, all samples were sectioned and subjected to the microtensile bond strength. For this test, the stick-shaped specimen was used, which has good acceptance, mainly because it does not require constrictions at the adhesive interface, for example, in the hourglass-shaped specimen. However, this type of specimen is more vulnerable to failures during the cutting process with the diamond disc, in addition to having sharp angles that concentrate more stresses.^21^ Failure modes were categorized into adhesive failure, cohesive natural tooth failure, cohesive typodont failure, and cohesive resin failure. The results demonstrate a similar pattern of bond strength values in MPa between natural and typodont teeth and predominance of the adhesive failure mode (79.9%). The results of the study show that there was no statistical difference in bond strength between the types of teeth; therefore, there was satisfactory adhesion. It is very important for the validation of the CS data that the adhesions between the substrates be well established.^13^ Because the typodont is made of epoxy resin, the surface was sandblasted with 50 µm aluminum oxide for 30 seconds. As shown by the results, sandblasting was effective, ensuring bond strength levels when associated with the universal adhesive system, compared to the enamel and dentin substrate. The single bond universal adhesive is composed of phosphate acid monomers (MDP) and silane, which favored the bond strength in typodont.

The use of typodont has advantages over natural teeth. The typodont model allows better geometry standardization and, as consequence, isolation of the effects of the resin composite. Typodont acquisition is also easy because it can be purchased from a typodont manufacturing company. Typodont may facilitate the testing of new materials because it does not require time to collect teeth and there is a greater power of sample standardization. Typodonts resemble natural teeth in terms of morphology but differ in their constitution, not accurately reflecting the properties of natural teeth. Typodont is made of epoxy resin, whose elastic modulus is 32 GPa. Before implementing the methodology, tests were carried out with different brands of typodont. Obviously, we found differences in their mechanical properties. The MOM typodont showed better elastic modulus results, similar to the dentin values. On the other hand, enamel and dentin are considered orthotropic and present variations in their values. The reported averages were 18.3 GPa^25^ for dentin and 84 GPa for enamel.^26^ The differences in these mechanical properties are clearly exhibited in the stress distributions observed in the finite element analysis (Figure 4). The main difference is that the stresses are lower in the typodont model (note the different stress scale for typodont and natural models). While natural teeth are concentrated in the cusp base region, typodont teeth are more concentrated at the restoration site (Figures 4B and 4C). This difference is expected due to the lower elastic modulus and higher compliance of the typodont teeth. Therefore, the typodont is not a good alternative when the factor under study is the evaluation of the stress distribution on the tooth structure because it does not reflect the natural characteristics of enamel and dentin. On the other hand, stresses on the composite and at the interfaces are easily comparable, but also with lower values for the typodont. This can also be explained by the differences in the elastic modulus and higher compliance of the typodont. Finite element analysis also expressed the differences in the stresses generated by the different composites, given that Filtek Z100 showed higher stress values, regardless of the type of tooth. In natural teeth, the lingual cusp concentrated more stress than the buccal cusp, corroborating the results presented by other authors.^16,24,27^These data can be explained by the fact that the lingual cusp has less morphological structure and the fact that the preparation weakens it.

## Conclusion

The CS of typodont teeth caused by different resin composites (conventional or low- shrinkage) showed a shrinkage stress effect during the restoration, similar to that of natural teeth. The use of typodont teeth for CS measurements using the strain gauge method is a viable alternative for polymerization stress analysis of resin composites.
